# Nitric Oxide and Hydrogen Sulfide in Higher Plants under Physiological and Stress Conditions

**DOI:** 10.3390/antiox8100457

**Published:** 2019-10-07

**Authors:** Francisco J. Corpas

**Affiliations:** Antioxidant, Free Radical and Nitric Oxide in Biotechnology, Food and Agriculture Group, Department of Biochemistry, Cell and Molecular Biology of Plants, Estación Experimental del Zaidín, CSIC, C/Profesor Albareda 1, E-18008 Granada, Spain; javier.corpas@eez.csic.es

Nitric oxide (NO) and hydrogen sulfide (H_2_S) are two gasotransmitters endogenously generated in plant cells. Each belongs to a family of related molecules called reactive nitrogen and sulfur species (RNS and RSS), respectively, which perform multiple functions in the physiology of plants. NO and H_2_S appear to share various plant tasks and are involved in a wide range of physiological processes, including seed germination, root architecture, plant growth and development, stomatal movement, senescence and fruit ripening, as well as in the mechanism of response to environmental stresses [1,2,3,4]. Their mechanism of action is mainly through protein posttranslational modifications, such as *S*-nitrosation, nitration and persulfidation, which affect the redox status and function of target proteins [5,6,7]. NO and H_2_S, which mediate several signaling networks, are key elements in the biochemistry and physiology of plants. Furthermore, increasing experimental data demonstrate crosstalk between these molecules and reactive oxygen species (ROS) metabolism. However, information on the plant cell metabolism of NO and H_2_S is still limited compared to that on ROS metabolism. A simple search in the PubMed database using the terms plant and the corresponding reactive species enables one to access all of the relevant information published in this field, wherein RNS and RSS account for only 23% and 1% of the total, as compared to 76% for ROS (Figure 1). 

Advances in scientific knowledge are made through the accumulation of information published in research papers. This special issue on “Nitric oxide (NO) and hydrogen sulfide (H_2_S) in higher plants under physiological and stress conditions” aims to provide up-to-date research in the area of NO, H_2_S and ROS metabolism. One review and six research papers have been brought together in this issue which offers new insights into the role played by these signaling molecules. The review by Kolbert et al. [8] provides a broad perspective on the interaction between NO and the phytohormone ethylene, which is either synergistic or antagonistic depending on the physiological process ranging from seed germination and development to senescence and fruit ripening, as well as the mechanism of response to stressful conditions. The authors point out that, while the NO signal cascade mainly takes place through protein posttranslational modifications, the effects of ethylene are initiated by a specific receptor. On the other hand, the six research papers study different aspects of the involvement of NO and H_2_S in beneficial interactions between microorganisms and plant roots, as well as in the ripening process of climacteric and non-climacteric fruits. Using genetic techniques, Fukusome et al. [9] analyze how the level of NO level regulated by heme-containing protein phytoglobin 1 (Glb1) beneficially affects nodule formation during invasions of *Lotus japoniscus* roots by *Mesorhizobium loti*, especially under hypoxic stress conditions triggered by flooding, and also demonstrate a concomitant reduction in ROS production. *Azolla pinnata* is a water fern characterized by a rapid root abscission phenomenon in response to certain environmental stimuli. The study by Yamasaki et al. [10] uses this model to analyze the RSS metabolism with the aid of l- and d-cysteine as the potential source of H_2_S, as well as a battery of new H_2_S donors including polysulfides. Their findings demonstrate that d-cysteine is a major substrate for H_2_S production in *Azolla pinnata* which is mediated by d-cysteine desulfhydrase activity, while the root abscission phenomenon is a good model for evaluating the efficiency of different H_2_S donors in aqueous solutions. The study by Chu-Puga et al. [11] shows that, during the ripening of non-climacteric sweet pepper (*Capsicum annuum* L) fruit, there is an increase in the content of lipid peroxidation, a marker of oxidative stress, which is associated with an increase in superoxide-generating respiratory burst oxidase homolog (Rboh) activity. Using in vitro assays, they also show that Rboh activity is inhibited by NO, peroxynitrite (ONOO^−^) and reduced glutathione (GSH), suggesting that this activity is modulated by posttranslational modifications, such as *S*-nitrosation, tyrosine nitration and glutathionylation, and that fruit ripening is associated with nitro-oxidative stress. Rodriguez-Ruiz et al. [12], who carried out a biochemical characterization of the peroxisomal antioxidant catalase in pepper fruits, found that this catalase has an atypical molecular mass, suggesting that, unlike the typical tetrameric catalase present in higher plants, it is a homodimer. By analyzing in vitro pepper catalase, the authors found that the activity of this antioxidant enzyme is negatively modulated by *S*-nitrosation and nitration. Lokesh et al. [13] study how the exogenous application of NO triggers polyamine accumulation in postharvest banana (*Musa acuminate*) fruit, apparently via the arginine-mediated route. Meanwhile, the study by Geng et al. [14] shows that H_2_S content is modulated by NO in peach (*Prunus persica*) fruit during cold storage, whose quality is adversely affected by the chilling injury phenomenon.

In summary, this special issue provides novel information on the complex relationship between H_2_S and NO which also affect the metabolism of ROS. It is worth noting that some research included in this issue focuses on plant species of agronomic interest, such as climacteric and no-climacteric fruits, whose ripening and quality can be affected during postharvest storage by the exogenous application of NO and/or H_2_S.

## Figures and Tables

**Figure 1:**
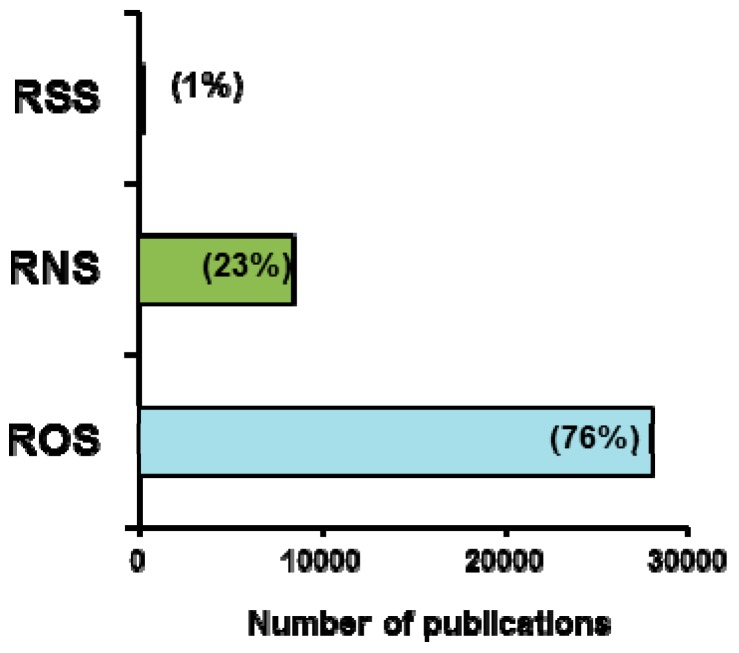
Number of publications related with reactive oxygen, nitrogen and sulfur species (ROS, RNS and RSS, respectively) and plants found in PubMed database.
